# Factors Associated With Control of Diabetes and Hypertension Among Patients Seen as Part of a Longitudinal Medical School Service-Learning Program From 2018-2019: An Exploratory Analysis

**DOI:** 10.7759/cureus.28225

**Published:** 2022-08-21

**Authors:** Raelynn Vigue, Waleem E Hernandez, Ashley L Ramirez, Grettel Castro, Noel C Barengo, David R Brown, Juan Ruiz-Pelaez

**Affiliations:** 1 Department of Translational Medicine, Herbert Wertheim College of Medicine, Florida International University, Miami, USA; 2 Department of Medical and Population Health Sciences Research, Herbert Wertheim College of Medicine, Florida International University, Miami, USA; 3 Department of Health Policy and Management, Robert Stempel College of Public Health, Florida International University, Miami, USA; 4 Department of Family Medicine, West Kendall Baptist Hospital, Miami, USA; 5 Department of Humanities, Health, and Society, Herbert Wertheim College of Medicine, Florida International University, Miami, USA; 6 KMC Senior Researcher, Kangaroo Foundation, Bogota, COL

**Keywords:** social determinant of health, socio demographic factors, public health care, hypertension control, diabetes mellitus type 2

## Abstract

Introduction

The Florida International University (FIU) Green Family Neighborhood Health Education Learning Program (NeighborhoodHELP) in Miami-Dade County serves communities impacted by adverse social determinants of health. This study identified sociodemographic factors affecting control of diabetes and hypertension among NeighborhoodHELP patients.

Methods

This non-concurrent cohort study evaluated NeighborhoodHELP patients who received care at mobile health centers (MHCs) utilizing de-identified data extracted from the MHCs' clinical quality metrics data set for the 2018-2019 fiscal year. A total of 143 eligible adults with diabetes and 222 adults with hypertension were identified. Condition control was defined as blood pressure ≤ 130 mmHg (systolic) and ≤ 80 mmHg (diastolic) or hemoglobin A1c (HbA1c) ≤ 7% (diabetes). Association with age, gender, ethnicity, marital status, language, service area, income per-capita, and medical student assignment was explored using logistic regression.

Results

The model showed decreased diabetes control likelihood among Haitian-Creole speakers (OR: 0.13; 95% CI: 0.02-0.75). Odds of diabetes control were greater in two discrete areas serviced by the program, one known as Hippocrates (OR: 4.9; 95% CI: 1.23-19.37) and the other Semmelweis (OR: 3.71; 95% CI: 1.07-12.83). Income greater than $10,000 increased hypertension control likelihood (OR: 2.22; 95% CI: 1.03-4.8).

Conclusions

Among NeighborhoodHELP patients, geographic region and language impact diabetes control, while income affects hypertension control. Further research is warranted to identify the role of other factors.

## Introduction

Miami-Dade County is an ethnically diverse community composed of 16.73% Black, 1.53% Asian, 5.92% of another race, and 75.60% White individuals, of which 90% are Hispanic. Within Miami-Dade County, 9.2% of the population are diagnosed with diabetes, and 32.7% of the population are diagnosed with hypertension [1]. Several health disparities exist within this community, in parallel with observed national trends. Minority groups, particularly Black individuals, suffer disproportionately from both diabetes and hypertension, with a prevalence of 15.4% and 27%, respectively [1]. Within Miami-Dade County, Black individuals experience increased rates of emergency room admissions, and morbidity and mortality associated with hypertension and diabetes [1]. Despite increasing awareness and medication use, Black patients continue to show lower rates of hypertension control when compared to non-Hispanic White patients [2].

Many of these disparities can be attributed to socioeconomic factors and related psychosocial stressors [3]. Management of hypertension and diabetes places a significant financial burden on patients and the health care system, while lack of control can lead to a decreased quality of life, increased stress, serious morbidity, and mortality. Ethnicity, socioeconomic status, and language are some of the many factors affecting patient care [4-5]. Additionally, marriage may also have protective health effects, with improved management of chronic conditions, medication adherence, and overall health outcomes [6-8]. Communication barriers and language discordance between patients and physicians also contribute to health disparities in minority populations, resulting in diminished continuity of care, physician-patient counseling, and patient compliance [9-11]. Low English proficiency has also shown an association with lower socioeconomic status, further perpetuating the existing disparity. Within Miami-Dade County, a majority of the population speaks Spanish (66%), with only 26% of the population solely speaking English. The remaining 7% speak Indo-European languages [1]. Thus, language concordance among patients and medical practitioners may have a substantial impact on medical outcomes among Miami-Dade residents.

The Florida International University (FIU) Green Family Neighborhood Health Education Learning Program (NeighborhoodHELP) is a novel public health program founded in 2010 and designed to engage medically underserved communities in Miami-Dade County while educating medical students on the social determinants of health [12]. Since its inception, NeighborhoodHELP has served nearly 6,000 patients through mobile health centers (MHCs) [12]. Beginning in their second year of medical school, FIU medical students take on the responsibility of leading an interdisciplinary team consisting of nursing, physician assistant, and social work students in the holistic care of an enrolled household. Through monthly contact and direct visits to patient homes, medical students are actively engaged in the medical care of their assigned household, while also addressing various socioeconomic challenges their patients may be facing, such as foreclosure, immigration status, and access to food. Through this program, uninsured patients may also receive free medical care from primary care providers at three MHCs that travel to assigned geographic areas in Miami-Dade County on alternating days throughout the week. The MHCs are fully equipped recreational vehicles with two patient rooms that provide an easily accessible location for patients with limited transportation and availability. The program is subdivided into four service areas: Semmelweis, Hippocrates, Pasteur, and Anderson. Semmelweis represents the communities of North Miami, North Miami Beach, and Little Haiti [13]. Hippocrates represents Miami Gardens [14], while Pasteur represents Opa-locka and South Miami [15]. Lastly, Anderson represents the communities of unincorporated northwest Dade, Hialeah, Hialeah Gardens, Miami Lakes, and Medley [16]. Medical students are assigned to a service area upon entry to medical school and provide care to an enrolled household in one of these areas.

There is very limited literature evaluating the subset of Miami-Dade County served by NeighborhoodHELP since its inception in 2010. This exploratory study sought to serve as the first of its kind in the evaluation of the impact of socioeconomic and sociodemographic factors on the control of chronic conditions among NeighborhoodHELP patients receiving care at the MHCs.

The purpose of this study was to identify which factors affect the management of hypertension and diabetes mellitus in patients being treated at the NeighborhoodHELP MHCs. It was hypothesized that low per capita income would show association with decreased likelihood of diabetes and hypertension control. Given the impact of language discordance on chronic condition management, lower odds of control of diabetes and hypertension was hypothesized among Spanish and Creole speakers. Lastly, lower odds of hypertension control were predicted among non-Hispanic Black patients due to existing data regarding lower rates of hypertension control among Black patients [2].

## Materials and methods

Study design and population 

This retrospective cohort study evaluated patients who received care at the NeighborhoodHELP MHCs between 2018 and 2019. Subjects were included if they were a registered participant in the NeighborhoodHELP program, at least 18 years of age, had a confirmed diagnosis of diabetes or hypertension, and had at least two visits to the MHCs during the measurement year or the year prior. Two separate anonymized limited data sets were constructed from the clinical quality metrics files for the NeighborhoodHELP MHCs for the 2018-2019 fiscal year, one each for diabetes and hypertension, respectively. Due to limited information regarding the presence of comorbid diabetes or hypertension in these de-identified data sets, patients were evaluated singularly as diabetes or hypertension patients. Patient data regarding illness severity, insulin dependence, and medical management regimens were not evaluated in this study.

Variables

The independent variables of this study were as follows: patient age, sex (biological male or female), ethnicity (Hispanic/Latino, non-Hispanic Black, non-Hispanic White), marital status (married, single, no longer married), language spoken (English, Spanish, Haitian-Creole), service area (Semmelweis, Hippocrates, Pasteur, Anderson, Out of student area), annual household per capita income (≤$10,000 or >$10,000), and assignment of a medical student (assigned, not assigned). Service areas represented a geographic region of South Florida serviced by the NeighborhoodHELP MHCs. These included Semmelweis (North Miami, North Miami Beach, and Little Haiti), Hippocrates (Miami Gardens), Pasteur (Opa-locka and South Miami), Anderson (unincorporated northwest Dade, Hialeah, Hialeah Gardens, Miami Lakes, and Medley), and areas lying outside of medical student assignment [13-16]. The outcome of interest was effective condition control. Diabetes control was defined as a hemoglobin A1c (HbA1c) level below 7% according to the last available measurement per American Diabetes Association (ADA) practice guidelines [17], while control of hypertension was defined as a systolic blood pressure less than or equal to 130 mmHg and a diastolic blood pressure less than or equal to 80 mmHg in concordance with American Heart Association (AHA) practice guidelines [18]. 

Statistical analysis

Analysis consisted of four steps utilizing STATA 14 software (StataCorp, College Station, TX). Three patients with diabetes mellitus were excluded from analysis due to missing hemoglobin A1c values or reporting errors. First, descriptive statistics were computed to profile the included samples of both diabetes and hypertension participants. Second, for each subsample (diabetes and hypertension), a bivariate analysis was performed to compare the distribution of diabetes and hypertension, respectively, according to levels of each of the independent variables being studied (age, sex, ethnicity, marital status, language spoken, service area, annual household income per capita, and medical student assignment). Chi-square statistics were computed for each bivariate comparison. Next, all potential predictors were tested for collinearity. Lastly, a multivariable analysis using a logistic regression model was conducted to evaluate the association between each one of the independent variables, and effective control of hypertension and diabetes. Statistical significance was determined utilizing an alpha of 0.05.

Ethical considerations

This study protocol was presented to the Florida International University Office of Research Integrity for review and approval. Because all data were already collected and fully de-identified, it was classified as Not Human Subject Research (NHSR) and was assigned IRB Protocol NHSR #IRB-20-0481.

## Results

**Figure 1:**
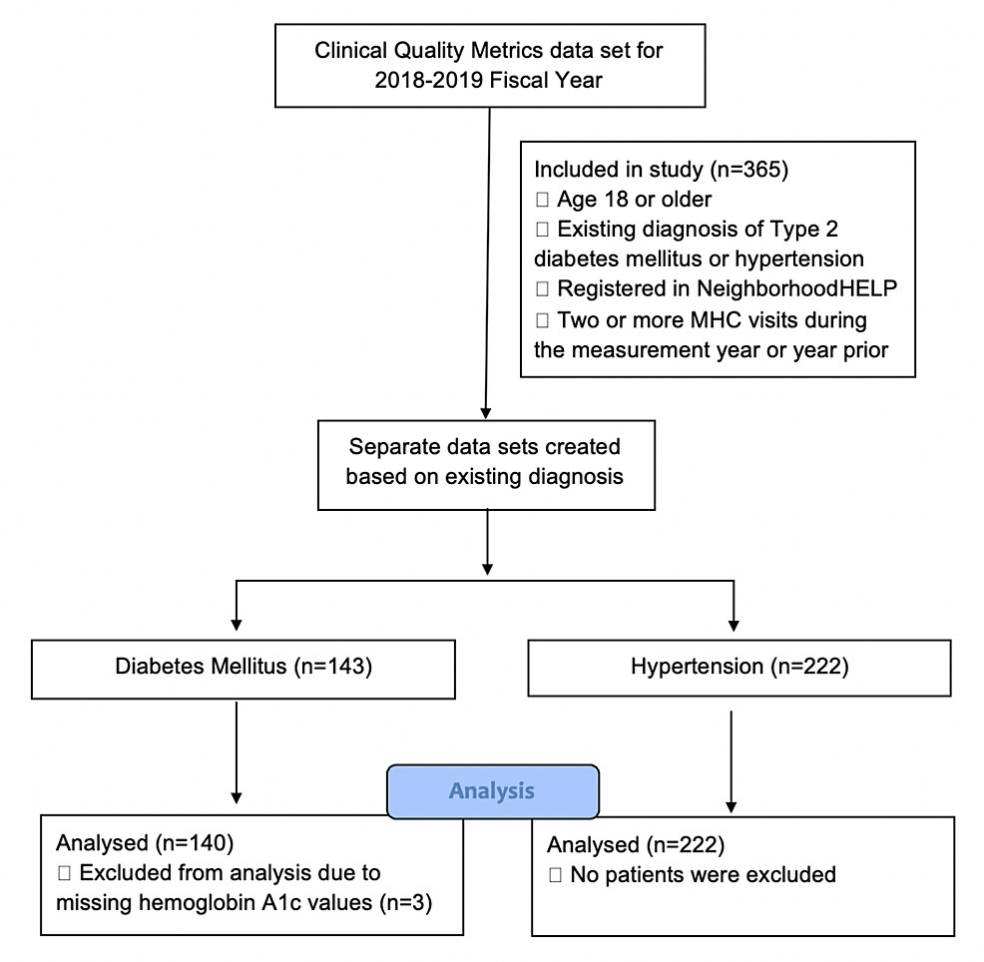
Sample of the study A pre-approved, de-identified data set including adult patients with two or more MHC visits during the measurement year or the year prior with a diagnosis of diabetes mellitus or hypertension was obtained from the MHC Clinical Quality Metrics data set for use in this study. MHC, mobile heath center

The total studied sample consisted of 143 adult patients with diabetes and 222 adult patients with hypertension who were enrolled in the NeighborhoodHELP program and had two or more MHC visits during the measurement year or the year prior. The sample was provided in a pre-approved, de-identified format compiled from the clinical quality metrics data set for the MHCs for the 2018-2019 fiscal year. Among the diabetic patients, three were excluded from analysis due to missing HbA1c values, leaving 140 patients in the diabetes subsample. The study design is depicted in Figure 1. In total, condition control was present at the time of most recent measurement in 70 (50%) diabetic participants and 152 (68.5%) participants with hypertension.

**Figure 2:**
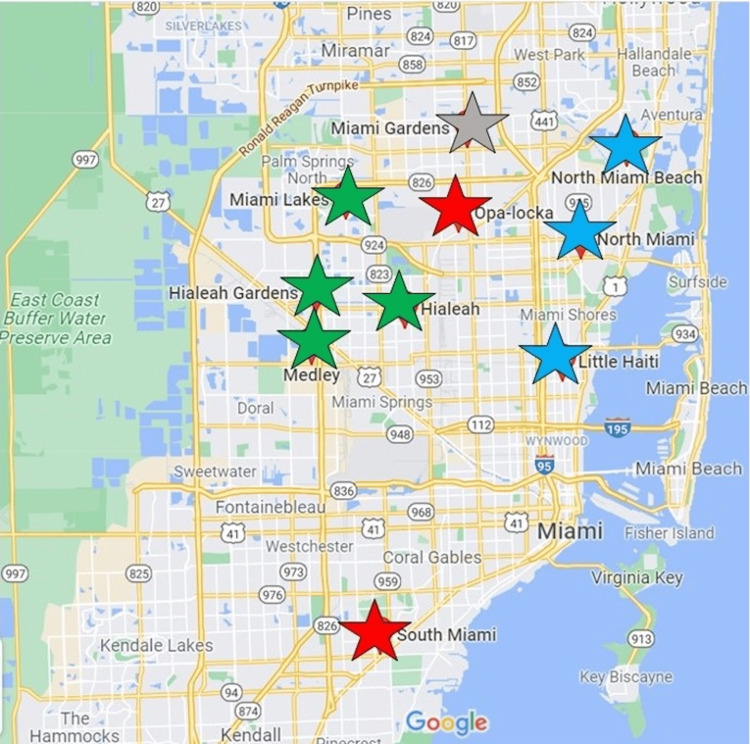
NeighborhoodHELP service areas Blue represents Semmelweis (North Miami, North Miami Beach, and Little Haiti), gray represents Hippocrates (Miami Gardens), red represents Pasteur (Opa-locka and South Miami), and green represents Anderson (unincorporated northwest Dade, Hialeah, Hialeah Gardens, Miami Lakes, and Medley) [13-16].

A map of the service areas with corresponding geographic locations in South Florida is depicted in Figure 2.

Diabetes mellitus

Participants with diabetes were on average over 50 years of age, predominantly female, and about 50% were Hispanic (Table 1). The other half were non-Hispanic Black patients, while only 3% were non-Hispanic White patients, and about 50% were married. The most common primary language spoken was English (50%), with 33% primarily speaking Spanish and 20% speaking Haitian Creole. Most of the patient population (89.1%) had a per capita income less than or equal to $10,000 per person and had a medical student assigned to their household.

**Table 1:** Characteristics of NeighborhoodHELP patients with diabetes ^a^Controlled diabetes was defined as HbA1c less than or equal to 7% according to the last available measurement. ^b^SD represents standard deviation. ^c^Service area represents a subset of NeighborhoodHELP by region of Miami-Dade County.

Patient Characteristics	N	%
Diabetes control^a^		
Uncontrolled	70	50
Controlled	70	50
Age, mean (SD^b^)	56.1 (8.8)	
Gender		
Female	91	65
Male	41	35
Race/ethnicity		
Hispanic or Latino	67	50.4
Non-Hispanic White	4	3
Non-Hispanic Black	62	46.6
Marital status		
Married	69	49.6
Single	46	33.1
No longer married	24	17.3
Language		
English	70	50
Haitian Creole	23	16.4
Spanish	47	33.6
Service area^c^		
Anderson	36	26.1
Hippocrates	31	22.5
Pasteur	22	15.9
Semmelweis	39	28.3
Out of student areas	10	7.2
Income per capita		
≤$10,000/person	125	89.1
>$10,000/person	15	10.9
Medical student assigned		
Yes	94	67.1
No	46	32.9

Among all variables examined in Table 2, only patient language demonstrated a statistically significant difference in the distribution of control of diabetes (p=0.01). The lowest proportions of control of diabetes were observed among Haitian Creole speakers (16%), followed by Spanish speakers (34%), and the highest levels of control occurred among English speakers (50%).

**Table 2:** Characteristics of NeighborhoodHELP patients with controlled diabetes mellitus according to HbA1c In this table, controlled diabetes was defined as HbA1c less than or equal to 7% according to the last available measurement, and HbA1c (Hemoglobin A1c) represents an average blood sugar over the last 2-3 months. The bolded value indicates statistically significant findings ^a^SD represents standard deviation. ^b^Service area represents a subset of NeighborhoodHELP by region of Miami-Dade County.

Characteristics	Uncontrolled		Controlled		p-Value
	N	%	N	%	
Age, mean (SD^a^)	55.8 (9.5)		56.4 (8.1)		0.69
Sex					0.59
Female	47	51.7	44	48.3	
Male	23	46.9	26	53.1	
Race/ethnicity					0.18
Hispanic or Latino	29	43.3	38	56.7	
Non-Hispanic White	2	50.0	2	50.0	
Non-Hispanic Black	37	59.7	25	40.3	
Marital status					0.6
Married	35	50.7	34	49.3	
Single	25	54.3	21	45.7	
No longer married	10	41.7	14	58.3	
Language					0.01
English	33	47.1	37	52.9	
Haitian Creole	18	78.3	5	21.7	
Spanish	19	40.4	28	59.6	
Service area^b^					0.69
Anderson	21	58.3	15	41.7	
Hippocrates	13	41.9	18	58.1	
Pasteur	11	50.0	11	50.0	
Semmelweis	20	51.3	19	48.7	
Out of Student Area	4	40.0	6	60.0	
income per capita					0.17
≤$10,000/person	64	52.0	59	48.0	
>$10,000/person	5	33.3	10	66.7	
Medical student assigned					0.72
Yes	46	48.9	48	51.1	
No	24	52.2	22	47.8	

Table 3 displays the unadjusted and adjusted associations between general characteristics of participants with diabetes and achieving condition control. Prior to multivariable adjustment, the only variable significantly associated with achieving control of diabetes was the patient’s spoken language. When compared with Spanish-speaking patients, those who spoke Haitian Creole had 81% lower odds of achieving diabetic control (OR: 0.19; 95% CI: 0.06-0.59). After fitting a multivariable logistic regression model including all variables listed in Table 3, language and service area also demonstrated independent associations with the odds of achieving diabetic control. When compared to Spanish speakers, Haitian Creole speakers had decreased odds of diabetes control (OR: 0.13; 95% CI: 0.02-0.75). As compared to patients from the Anderson service area, those from Hippocrates (Miami Gardens) and from Semmelweis (North Miami, North Miami Beach, Little Haiti) had 4.9 (95% CI: 1.07-12.83) and 3.7 (95% CI: 1.23-19.37) higher odds of achieving diabetic control, respectively.

**Table 3:** Unadjusted and adjusted associations between NeighborhoodHELP patient characteristics and diabetes mellitus control Controlled diabetes was defined as HbA1c less than or equal to 7% according to the last available measurement. The bolded values indicate statistically significant findings ^a^Service area represented a subset of NeighborhoodHELP by region of Miami-Dade County.

Characteristics	Unadjusted		Adjusted	
	OR (95% CI)	p-Value	OR (95% CI)	p-Value
Age	1	0.69	1.02 (0.98-1.07)	0.33
Sex				
Female	Reference		Reference	
Male	1.2 (0.60-2.42)	0.6	1.27 (0.54-2.99)	0.59
Race/ethnicity				
Hispanic or Latino	Reference		Reference	
Non-Hispanic White	0.76 (0.1-5.75)	0.79	0.65 (0.07-6.34)	0.71
Non-Hispanic Black	0.52 (0.26-1.04)	0.06	0.76 (0.23-2.43)	0.64
Marital status				
Married	Reference		Reference	
Single	0.86 (0.41-1.83)	0.7	0.87 (0.36-2.12)	0.77
No longer married	1.44 (0.56-3.68)	0.45	1.52 (0.5-4.68)	0.46
Language				
English	0.76 (0.36-1.61)	0.47	0.56 (0.17-1.85)	0.34
Haitian Creole	0.19 (0.06-0.59)	0.004	0.13 (0.02-0.75)	0.02
Spanish	Reference		Reference	
Service area^a^				
Anderson	Reference		Reference	
Hippocrates	1.94 (0.73-5.13)	0.18	4.9 (1.23-19.37)	0.02
Pasteur	1.4 (0.48-4.07)	0.54	2.7 (0.72-10.48)	0.14
Semmelweis	1.33(0.53-3.31)	0.54	3.71 (1.07-12.83)	0.04
Out of student area	2.1 (0.50-8.76)	0.31	3.18 (0.55-18.33)	0.2
Income per capita				
≤$10,000/person	Reference		Reference	
>$10,000/person	2.17 (0.70-6.72)	0.18	3.74 (0.99-14.13)	0.05
Medical student assigned				
Yes	Reference		Reference	
No	0.99 (0.43-1.78)	0.72	1.03 (0.45-2.37)	0.94

Hypertension

Participants with hypertension averaged over 50 years of age and were predominantly female (Table 4). Around 50% were Hispanic, with the other 50% composed primarily of non-Hispanic Black patients, while only 7.8% were non-Hispanic White patients. About 50% of the subjects were married. Nearly 40% spoke Spanish, while almost 20% spoke Haitian Creole and the remaining 50% spoke English. Most hypertensive patients had a medical student assigned to their household and had a per capita income of greater than $10,000 per person (81.8%).

**Table 4:** Characteristics of NeighborhoodHELP patients with hypertension ^a^Controlled hypertension was defined as a systolic blood pressure less than or equal to 130 mmHg and a diastolic blood pressure less than or equal to 80 mmHg. ^b^SD represents standard deviation. ^c^Service area represents a subset of NeighborhoodHELP by region of Miami-Dade County.

Patient Characteristics	N	%
Hypertension control^a^		
Uncontrolled	70	31.5
Controlled	152	68.5
Age, mean (SD^b^)	58.5 (9.2)	
Gender		
Female	142	64
Male	80	36
Race/ethnicity		
Hispanic or Latino	107	50.2
Non-Hispanic White	8	7.8
Non-Hispanic Black	98	46
Marital status		
Married	112	50.9
Single	66	30
No longer married	42	19.1
Language		
English	91	41
Haitian Creole	42	18.9
Spanish	89	40.1
Service area^c^		
Anderson	59	26.8
Hippocrates	45	20.4
Pasteur	32	14.6
Semmelweis	65	29.6
Out of student areas-Baptist	19	8.6
Income per capita		
≤$10,000/person	40	18.2
>$10,000/person	180	81.8
Medical student assigned		
Yes	156	70.3
No	66	29.7

Table 5 displays the incidence of proper control of hypertension according to the general characteristics of participants. Statistically significant associations with hypertension control were found for language spoken and per capita income. No other significant associations were identified.

**Table 5:** Characteristics of NeighborhoodHELP patients with controlled hypertension( according to systolic and diastolic blood pressure Controlled hypertension was defined as a systolic blood pressure less than or equal to 130 mmHg and a diastolic blood pressure less than or equal to 80 mmHg. The bolded values indicate statistically significant findings ^a^SD represents standard deviation. ^b^Service area represents a subset of NeighborhoodHELP by region of Miami-Dade County.

Characteristics	Uncontrolled		Controlled		p-Value
	N	%	N	%	
Age, mean (SD^a^)	58.4 (10.7)		58.6 (9.0)		0.92
Sex					0.59
Female	43	30.3	99	69.7	
Male	27	33.8	53	66.2	
Race/ethnicity					0.07
Hispanic or Latino	27	25.2	80	74.8	
Non-Hispanic White	2	25	6	75	
Non-Hispanic Black	39	39.8	59	60.2	
Marital status					0.21
Married	34	30.4	78	69.6	
Single	26	39.4	40	60.6	
No longer married	10	23.8	32	76.2	
Language					0.04
English	30	33	61	67	
Haitian Creole	19	45.2	23	54.8	
Spanish	21	31.5	152	68.5	
Service area^b^					0.98
Anderson	18	30.5	41	69.5	
Hippocrates	13	28.9	32	71.1	
Pasteur	11	34.4	21	65.6	
Semmelweis	22	33.8	43	66.2	
Out of student area	6	31.6	13	68.4	
Income per capita					0.006
≤$10,000/person	20	50	20	50	
>$10,000/person	50	27.8	130	72.2	
Medical student assigned					0.23
Yes	53	34	103	66	
No	17	25.8	49	74.2	

Table 6 depicts the unadjusted and adjusted associations between general characteristics of hypertensive participants and achieving blood pressure control. Prior to multivariable adjustment, the variables significantly associated with achieving control of hypertension were language spoken by the patient, race/ethnicity, and per capita income. When compared to Spanish-speaking hypertensive patients, those who spoke Haitian Creole had 63% lower odds of achieving hypertensive control (OR: 0.37; 95% CI: 0.17-0.82). When compared to Hispanic patients, non-Hispanic black patients had 49% lower odds of hypertensive control (OR: 0.51; 95% CI: 0.28-0.93). In addition, patients with a per capita income greater than $10,000 per person were 2.6 times more likely to achieve hypertensive control (OR: 2.6; 95% CI: 1.29-5.24). After fitting a multivariable logistic regression model including all variables listed in Table 6, only per capita income demonstrated an independent association with hypertension control. Once again, when compared to individuals falling at or below a per capita income of $10,000, individuals with a per capita income greater than $10,000 exhibited 2.6 times greater odds of control (OR: 2.6; 95% CI: 1.29-5.24).

**Table 6:** Unadjusted and adjusted associations between NeighborhoodHELP patient characteristics and hypertension control Controlled hypertension was defined as a systolic blood pressure less than or equal to 130 mmHg and a diastolic blood pressure less than or equal to 80 mmHg. The bolded values indicate statistically significant findings ^a^SD represents standard deviation. ^b^Service area represents a subset of NeighborhoodHELP by region of Miami-Dade County.

Characteristics	Unadjusted		Adjusted	
	OR (95% CI)	p-value	OR (95% CI)	p-value
Age, mean (SD^a^)	1 (0.97-1.03)	0.92	1 (0.97-1.03)	0.96
Sex				
Female	Reference		Reference	
Male	0.85 (0.47-1.53)	0.59	0.89 (0.47-1.7)	0.72
Race/ethnicity				
Hispanic or Latino	Reference		Reference	
Non-Hispanic White	1.01 (0.19-5.32)	0.988	1.29 (0.18-9.12)	0.8
Non-Hispanic Black	0.51 (0.28-0.93)	0.027	0.80 (0.25-2.55)	0.71
Marital status				
Married	Reference		Reference	
Single	0.67 (0.35-1.27)	0.22	0.91 (0.43-1.94)	0.35
No longer married	1.49 (0.62-3.16)	0.42	1.19 (0.11-2.13)	0.71
Language				
English	0.63 (0.33-1.21)	0.165	0.72 (0.23-2.27)	0.58
Haitian Creole	0.37 (0.17-0.82)	0.013	0.49 (0.11-2.13)	0.34
Spanish	Reference		Reference	
Service area^b^				
Anderson	Reference		Reference	
Hippocrates	1.08 (0.46-2.52)	0.86	1.5 (0.48-4.33)	0.68
Pasteur	0.84 (0.34-2.09)	0.71	0.87 (0.30-2.48)	0.79
Semmelweis	0.86 (0.40-1.83)	0.69	1.34 (0.53-3.42)	0.54
Out of student area	0.96 (0.31-2.90)	0.93	0.74 (0.2-2.78)	0.66
Income per capita				
≤$10,000/person	Reference		Reference	
>$10,000/person	2.6 (1.29-5.24)	0.01	2.22 (1.03-4.8)	0.04
Medical student assigned				
Yes	Reference		Reference	
No	1.48 (0.78-2.82)	0.23	1.56 (0.74-3.29)	0.24

## Discussion

Among patients served at the NeighborhoodHELP MHCs in the 2018-2019 fiscal year, primary language spoken and geographic location impacted control of diabetes, while per capita income impacted control of hypertension. The service areas of Miami Gardens (Hippocrates) and North Miami/North Miami Beach/Little Haiti (Semmelweis) showed a statistically significant increased likelihood of diabetes control, while speaking Haitian Creole as a primary language is associated with poorer odds of diabetes control when compared to Spanish-speaking patients. Among hypertensive individuals served by the NeighborhoodHELP MHCs, 81.8% have a per capita income greater than $10,000, which is aligned with the observed proportion of participants with adequate hypertension control (68.5%).

The impact of language spoken on likelihood of diabetes control is consistent with current literature regarding language discordance between patients and physicians and decreased glycemic control [19]. However, the available literature explores the impact of language discordance between Spanish and English speakers with limited evaluation of the impact of Haitian Creole and English discordance. This study is unique in that it evaluates control of diabetes and hypertension among English, Spanish, and Haitian Creole speakers. Most primary care providers working on MHCs speak both English and Spanish, which could contribute to the absence of difference in odds of diabetes control between English and Spanish speakers. Limited access to Haitian Creole-speaking providers within NeighborhoodHELP may have contributed to decreased likelihood of diabetes control among Haitian Creole-speaking patients. Additionally, it is possible that these patients may carry associated cultural factors and linguistic risk factors, such as dietary practices, cultural attitudes toward medicine, cultural and linguistic isolation, and health literacy that may impact control of diabetes [20-22].

No association was identified between marital status and control of either diabetes or hypertension. This contrasts with a 2010 study completed in an ethnically diverse population of type 2 diabetics in southern California, which revealed that married individuals experienced better social support contributing to improved health behaviors and control of chronic conditions [8]. This discrepancy may be present secondary to variability in population characteristics between South Florida and Southern California, although limited statistical power could also account for this discrepancy with the findings.

In addition, this study did not identify differences in achievement of control between men and women. This diverges from the findings of a 2006 meta-analysis that showed higher morbidity and mortality in female diabetic patients when compared to males, suggesting that adequate control of diabetes was impacted by patient sex [19].

It is unclear why the Hippocrates and Semmelweis service areas exhibited greater odds of diabetes control, as each region receives access to the same NeighborhoodHELP programming and resources. In addition, Hippocrates has the largest Non-Hispanic Black population, while Anderson has the largest Hispanic population. Thus, ethnic and linguistic factors may have contributed to these differences. Direct evaluation of sociodemographic features of patients within each service area may provide further insight into why this disparity exists. Management of chronic conditions can be costly, including access to healthy food, medications, and environments allowing for safe exercise. Thus, it seems logical that a per capita income greater than $10,000 per person confers an increased likelihood of hypertension control. Ultimately, future investigation is needed to explore the proposed mechanisms behind the findings of this study.

A borderline significant association was identified between per capita income and diabetes control (OR: 3.74; 95% CI: 0.99-14.13; p-value = 0.052). Within the study population, 89.1% of diabetic individuals fall below this line; therefore, this borderline finding merits the need for a study with a larger sample size and carefully sampled ethnic groups. It is also important to note that due to Miami’s multicultural population and NeighborhoodHELP’s focus, this study consisted of a small population of non-Hispanic White patients; thus, much of the disparity was assessed among different minority groups. Further investigation in comparison to a primarily non-Hispanic White population may provide additional perspective on disparities impacting NeighborhoodHELP patients.

A major limitation of this study is the small sample size due to the unique patient population being studied. It is possible that a limited study power could have underestimated the impact of the independent variables on control of diabetes and hypertension. Thus, repeated analysis utilizing a larger sample size is needed. In addition, several potentially influential independent variables were not available for inclusion in this study, such as duration of disease, severity of disease at enrollment into NeighborhoodHELP, educational level, health literacy, cultural attitudes, and access to healthy food and medications. Determination of cutoff values for diabetes and hypertension control presented an additional challenge in analysis. While standardized practice guidelines were utilized in this study, clinically acceptable HbA1c and blood pressure readings vary depending on patient characteristics and comorbidities. This study may be further strengthened by identification of comorbid diabetes among hypertension patients and appropriate adjustment of systolic and diastolic blood pressure cutoffs for determination of control. In addition, data regarding classification of diabetes severity (i.e. insulin-dependent versus not insulin-dependent) were not available for consideration in this study. However, this could have contributed to diabetes control likelihood and should be evaluated in future studies.

## Conclusions

Special consideration of socioeconomic and geographic factors should be applied to the care of NeighborhoodHELP patients seen at the MHCs. The results of this study may be immediately translated to clinical practice via implementation of targeted public health programming directed at the identified disparities. Several proposed interventions include improved access to Creole-speaking health professionals, development of educational materials in Haitian Creole, and in-depth comparison of each service area to identify areas for improvement. NeighborhoodHELP began serving the Miami-Dade community in September 2010 and has served nearly 6,000 patients through MHCs. While this study evaluated data compiled from the 2018-2019 fiscal year, continued analysis of data gathered from future years could contribute valuable insight into the limited available current literature and would provide the opportunity for ongoing evaluation of program effectiveness. In addition, the framework of this study may be applied to future analysis of the impact of sociodemographic factors on chronic condition control among NeighborhoodHELP patients. Further investigation is merited utilizing a larger sample size to evaluate alternative independent variables, including comorbidities, severity of disease at entrance into NeighborhoodHELP, health literacy, and access to affordable healthy food and medications.
